# Global Transcriptome Profile of *Cryptococcus neoformans* during Exposure to Hydrogen Peroxide Induced Oxidative Stress

**DOI:** 10.1371/journal.pone.0055110

**Published:** 2013-01-28

**Authors:** Rajendra Upadhya, Leona T. Campbell, Maureen J. Donlin, Rajeev Aurora, Jennifer K. Lodge

**Affiliations:** 1 Department of Molecular Microbiology, Washington University School of Medicine, Saint Louis, Missouri, United States of America; 2 Edward A. Doisy Department of Biochemistry and Molecular Biology, Saint Louis University School of Medicine, Saint Louis, Missouri, United States of America; 3 Department of Molecular Microbiology and Immunology, Saint Louis University School of Medicine, Saint Louis, Missouri, United States of America; Stony Brook University, United States of America

## Abstract

The ability of the opportunistic fungal pathogen *Cryptococcus neoformans* to resist oxidative stress is one of its most important virulence related traits. To cope with the deleterious effect of cellular damage caused by the oxidative burst inside the macrophages, *C. neoformans* has developed multilayered redundant molecular responses to neutralize the stress, to repair the damage and to eventually grow inside the hostile environment of the phagosome. We used microarray analysis of cells treated with hydrogen peroxide (H_2_O_2_) at multiple time points in a nutrient defined medium to identify a transcriptional signature associated with oxidative stress. We discovered that the composition of the medium in which fungal cells were grown and treated had a profound effect on their capacity to degrade exogenous H_2_O_2_. We determined the kinetics of H_2_O_2_ breakdown by growing yeast cells under different conditions and accordingly selected an appropriate media composition and range of time points for isolating RNA for hybridization. Microarray analysis revealed a robust transient transcriptional response and the intensity of the global response was consistent with the kinetics of H_2_O_2_ breakdown by treated cells. Gene ontology analysis of differentially expressed genes related to oxidation-reduction, metabolic process and protein catabolic processes identified potential roles of mitochondrial function and protein ubiquitination in oxidative stress resistance. Interestingly, the metabolic pathway adaptation of *C. neoformans* to H_2_O_2_ treatment was remarkably distinct from the response of other fungal organisms to oxidative stress. We also identified the induction of an antifungal drug resistance response upon the treatment of *C. neoformans* with H_2_O_2_. These results highlight the complexity of the oxidative stress response and offer possible new avenues for improving our understanding of mechanisms of oxidative stress resistance in *C. neoformans*.

## Introduction

*Cryptococcus neoformans* is pathogenic basidiomycetous yeast with a ubiquitous worldwide distribution. It exists primarily as an environmental organism associated with soil and is known to have a particular association with bird guano [1]. However, *C. neoformans* is also an important opportunistic pathogen that causes invasive fungal infections and is responsible for about 1 million cases and 700,000 mortalities annually [2]. Most of the infections have been reported in patients with immune deficiencies, especially those with AIDS, but also in non-HIV immune compromised patients. The respiratory tract is the main portal of entry and the lung is the primary site of infection, though serious infections involving the central nervous system are common.

In the mammalian host, cell mediated immunity based on phagocytic cells is crucial to counteract fungal infections. Macrophages, neutrophils and other phagocytic cells generate potent reactive oxygen (ROS) and nitrogen (RNS) species that are toxic to most fungal and bacterial pathogens and cause damage to their DNA, protein and lipids. ROS and RNS are implicated in the killing of fungal pathogens such as *Aspergillus fumigatus*, *Candida albicans*, and *C. neoformans* by host immune cells [3], [4], [5]. Most of these conclusions are based on the positive correlation between resistance of the wild type and sensitivity of a specific deletion strain to oxidative stress *in vitro* and the corresponding respective virulence and avirulence phenotypes of these strains in a murine cryptococcosis model [6].

An important virulence related trait of *C. neoformans* is its ability to survive inside phagocytic cells. It not only resists killing by macrophages after phagocytosis, but can continue to replicate by budding within this environment and subsequently exit the macrophage without causing host cell lysis [7], [8], [9], [10], [11]. The ability of *C. neoformans* to survive and thrive inside this harsh environment suggests it must have mechanisms not only to neutralize the reactive molecular species to which it is exposed within the macrophages but also to repair the cellular damages caused by the oxidative and nitrosative stresses.

In *C. neoformans,* a number of genes coding for enzymes of antioxidant defense systems have been shown to be important for both *in vitro* oxidative stress resistance and also for *in vivo* pathogenesis [12]. Foremost among these is the highly conserved peroxiredoxin, Tsa1, which is induced under oxidative stress and is essential for *C. neoformans* resistance to peroxide stress [6]. The downstream components of the thioredoxin system, represented by the thioredoxins Trx1 and Trx2 and thioredoxin reductase Trr1, are involved in the reduction and recycling of the oxidized, inactive form of peroxiredoxin [13]. In *C. neoformans*, deletion of these genes renders them sensitive to oxidative stress, albeit with a less severe phenotype than that of a *tsa1*Δ strain, and decreased survival in macrophage culture conditions [14], [15]. *C. neoformans* also contains two glutathione peroxidases Gpx1 and Gpx2, both of which respond differently to various stressors; with only Gpx2 induced in response to H_2_O_2_ generated oxidative stress. Furthermore, both *GPX1* and *GPX2* deletion mutants were only mildly sensitive to oxidant killing by macrophages and exhibited no effect on virulence in a murine model [16]. Other enzymes that have been shown to play a role in oxidative stress resistance in *C. neoformans* include a cytosolic copper-zinc superoxide dismutase (Sod1) [17], the mitochondrial manganese superoxide dismutase (Sod2) [18], cytochrome c peroxidase (Ccp1) [19] and the alternative oxidase (Aox1) [20].

Whole genome microarray studies of *C. neoformans* experiencing oxidative stress have been reported in which the authors either used fungal cells engulfed by macrophages or grown in the nutrient rich YPD medium and treated with exogenous H_2_O_2_ [21], [22]. The magnitude of the transcriptional response was much weaker in the cells residing inside the macrophages compared to the robust transcriptional response observed in the cells treated with exogenous H_2_O_2_ in YPD medium, suggesting that the environment has a direct effect on the transcriptional response. This may also be due to the differences in the concentration of ROS released by the exogenous application of stress agent compared to that released inside the macrophages. Gene expression studies of oxidative stress resistance in a number of other fungal organisms such as *S. cerevisiae*, *Schizosaccharomyces pombe* and *Candida sp* have been published [23], [24], [25], [26]. They indicate that the sensitivity of the organisms to different concentrations of H_2_O_2_ and the magnitude of the elicited cellular response was highly dependent on the composition of the medium in which the cells were grown and treated. For example, *S. pombe* triggered different signaling networks mediated either by Pap1 or by Sty1 depending on the concentration of H_2_O_2_ used for the treatment [27]. Pap1 was more sensitive to H_2_O_2_ than the Sty1-mediated pathway and was responsible for inducing an adaptive response. This was shown by an induction of Pap1 with an extracellular concentration of 0.2 mM H_2_O_2_, whereas H_2_O_2_ concentrations above 0.2 mM failed to trigger this activation.

In a study of oxidative stress and aging in *S. cerevisiae*, the authors reported different degrees of sensitivity of yeast cells to external H_2_O_2_ when grown in different media [28]. In this study, there was a dramatic increase in the resistance of yeast cells growing in YNB in the presence of galactose and required the use of 10 mM of H_2_O_2_ for gene induction, even though the same strains grown in the presence of glucose exhibited 70% death when treated with 1.5 mM H_2_O_2_. The pathogenic fungi *C. glabrata* has shown to be resistant to up to 40 mM of H_2_O_2_, while *C. albicans* was found to be sensitive at this concentration of H_2_O_2_ [29]. Another pathogenic fungi, *A. fumigatus,* continued to grow during two-hour incubations with 5 mM H_2_O_2_ and tolerated up to 30 mM H_2_O_2_ [30], [31].

In the context of pathogenic fungi and phagocytosis, the concentration of H_2_O_2_ inside the phagosome during the oxidative burst is not precisely known, however multiple reports suggest that the effective H_2_O_2_ concentration may reach hundreds of micro molar [32], [33]. Additionally, the usable nutrient composition of the phagosome is essentially unknown, but it is likely to be a nutrient-limited environment with the lumen of the phagosomes reported to acidify to a final pH of ∼ 4.8 during maturation [34]. To more closely mimic *in vivo* conditions, we chose to treat *C. neoformans* cells with H_2_O_2_ in a nutrient limited YNB medium at pH 4.0. To stress the cells and avoid the induction of an overwhelming apoptotic death response, cells were treated with a concentration of H_2_O_2_ that resulted in the killing of ∼ 15% of initial cell population or lethal dose (LD∼15) which was determined via H_2_O_2_-generated oxidative stress death curves. These curves dictated the use of 1 mM H_2_O_2_ for treatment. By quantitatively measuring the concentration of H_2_O_2_ in the culture during the treatment, we discovered that within 30 minutes of incubation, all of the H_2_O_2_ was completely degraded from the medium. Therefore, we subjected RNA samples isolated from the cells at 5, 15, 30, 45 and 60 minutes post H_2_O_2_ treatment to microarray hybridization. We used a custom designed *C. neoformans* serotype A array using the predicted ORFs in the H99 (serotype A) *C. neoformans* genome identified by the Broad Institute genome sequencing project (http://www.broadinstitute.org/annotation/genome/cryptococcus_ neoformans/FeatureSearch.html). To facilitate the identification of underlying biological processes from the gene expression dataset, we generated a new gene ontology gene association file for *C. neoformans* genome. Herein, we report a global time-resolved genomic expression pattern of *C. neoformans* and identified over-represented biological processes which may point to potential mechanisms by which the fungus detects and destroys the oxidative stressor as well as repairs and recovers from the damage caused by the oxidative stress.

## Materials and Methods

### Strains, Media and Reagents

*C. neoformans* serotype A strain KN99α was used throughout this experiment. Cells were grown on rich medium, YPD (1% yeast extract, 2% Bacto peptone, and 2% dextrose), or minimal medium, YNB, pH 4.0 (6.7 g/liter yeast nitrogen base without amino acids plus 20 g/liter dextrose and 25 mM sodium succinate at pH 4.0). Solid media contained 2% Bacto agar. Antimycin-A from *Streptomyces* sp. (Cat No A8674), Carbonyl cyanide 4-(trifluoromethoxy) phenylhydrazone (FCCP) (Cat No -C2920), Salicylhydroxamic acid (SHAM) (Cat No -S607) and hydrogen peroxide solution (Cat No H1009) all were purchased from SIGMA-ALDRICH, St Louis, MO, USA. Estimation of H_2_O_2_ was performed using OxiSelect H_2_O_2_ assay kit from Cell Biolabs, San Diego, CA, USA following the protocol supplied with the reagent. Three independent experiments were carried out to calculate the standard error which is indicated by the error bars in the figures.

### Determination of Stressor Concentration by Survival Curve

Exponentially growing cells (OD_650_ = 1.5) were treated with various concentrations of H_2_O_2_ in a shaking 30°C incubator. Aliquots were taken at various time points, cells pelleted by centrifugation at 4°C. Cell pellet was washed two times with cold PBS and finally resuspended in PBS for plating on solid YPD and incubated at 30°C. Colony forming units (CFU) were counted after 2 days.

### Hydrogen Peroxide Sensitivity Tests

Overnight cultures were diluted to OD_650_ = 1.0. Ten times serially diluted cell suspension was spotted on various plates containing either the individual stressor or their combinations. Plates were incubated at 30°C and observed on different days.

### Custom *C. neoformans* Serotype A Microarray Development

Of the 7239 predicted ORFs in the H99 (serotype A) *C. neoformans* genome identified through the Broad Institute *C. neoformans* genome sequencing project, 6,932 probes were designed. The remaining 307 genes were either identical paralogs or had primarily low complexity sequences. Single 60-mer oligonucleotide probes were designed for each of the open reading frames and were duplicated on each microarray to provide an estimate of intra array variance. The arrays were fabricated by Agilent Technologies, Santa Clara, CA.

### Cell Preparation and Treatment

Two-day-old cultures of *C. neoformans* KN99α were used to inoculate three bioreplicates into YNB pH 4.0 media and incubated at 30°C with shaking (200 rpm) until the cells were in mid-log phase (OD_650_ ∼1.5–2.0). Each biological replicate culture was split into two cultures and H_2_O_2_ was added to one of each of these two cultures to a final concentration of 1 mM. Cultures were incubated for 60 minutes and sampled at 5, 15, 30, 45 and 60-minute intervals. Sodium citrate (50 mM final concentration) was added to control and test samples to rapidly halt the hydrogen peroxide stress. Cells were collected by centrifugation at 1800 *g* for 5 minutes and washed once with sterile phosphate buffered saline. The washed cell pellets were flash frozen and lyophilized. Lyophilized cells were stored at –80°C.

### Isolation of Total RNA

RNA was isolated using the Agilent Total RNA Isolation (Protocol for Yeast) (Agilent Technologies, Palo Alto, CA.) according to the manufacturer’s instructions with the following modifications. The lyophilized pellet (approximately 1×10^8^ cells) was vortexed with 0.5 g of 1 mm glass beads (Biospec, Inc.) for 5 min., followed by the addition of 600 uL lysis solution (kit supplied) and vortexed for a further 5 min. Disrupted cells were centrifuged at 16 000 *g* for 3 min. and the supernatant transferred to a pre-filtration column. Rest of the procedures was carried out as described in the Manufacturer’s protocol until the final step. Isolated RNA was treated with RNase free DNase. RNA was quantified using a Nanodrop ND-1000 (Nanodrop Technologies, Wilmington, DE.). The quality of purified RNA was assessed on an Agilent 2100 Bioanalyzer (Agilent Technologies, Santa Clara, CA.).

### Labeling of Total RNA

Total RNA was directly fluorescently labeled with Cy3 or Cy5 using the Micromax ASAP RNA labeling kit (Perkin Elmer, Inc, Wellesley, MA.) according to manufacturer’s instructions with the following modifications. All reaction component volumes were doubled with the exception of RNA concentration resulting in a final reaction mix volume of 40 uL. After the addition of Stop buffer, to remove any unincorporated dye, the reaction mix volume was brought up to 100 uL with nuclease-free water followed by the addition of 3 volumes of 100% nuclease-free ethanol. This 400 uL reaction volume was then applied to an RNeasy Mini Kit column (Qiagen, Valencia, CA., USA) and processed according to manufacturer’s instructions (Yeast RNA isolation protocol, starting from RW1 wash). Dye incorporation was quantitated using a Nanodrop ND-1000 (Nanodrop Technologies) by measuring emission wavelength at 570 nm (Cy3) and 670 nm (Cy5). Labeled-RNA concentration and dye incorporation were used to determine the RNA labeling specific activity (pmol/ug). Labeled and unlabeled RNA were combined to adjust the sample specific activity to the empirically determined intensities of 40 and 45 pmol/ug for Cy3 and Cy5 labeled samples, respectively. Unstressed Cy3-labeled and 1 mM H_2_O_2_ stressed Cy5-labeled RNA were combined and brought to near-dryness in a vacuum centrifuge before being resuspended in 19 uL nuclease-free water and stored at –80°C.

### Hybridization to Microarrays

Microarray hybridization and scanning were preformed according to Agilent Microarray processing specifications (MO gene LLC, St Louis, MO. USA).

### Microarray Analysis

The LOESS balanced data from the Feature Extraction was used to assess replication consistency across biological and process replicates using a linear ANOVA model that considers all 13,864 probes on the array and data for all the replicates, to determine the significance of differential expression [35]. y_rijk_ = α+A_i_+G_k_+(AG)_ik_+D_l_+α_rijk_; where y_rijk_ is the logarithm of the r^th^ replicate model-based expression value of gene k at the i^th^ time point with the j^th^ treatment (i = 1, 2, 3, 4; j = 1, 2; k = 1,…, 41, 174). This model accounts for variance in genes (G), arrays (A), and array plus gene; α term accounts for error.

### Quantitative Real Time PCR

Archive RNA extracted for the microarray experiments was used to make first-strand cDNA using the First-Strand cDNA synthesis kit for reverse transcription-PCR (Roche). This cDNA was used as template for real-time PCR analysis using SsoFast SYBR green PCR reagents (Biorad) according to the manufacturer’s recommendations. A BioRad CFX96 thermal cycler was programmed with the following two step PCR cycles: 5 s at 98°C, 5 s at 60°C, and a plate read was repeated in the second step for a total of 40 cycles. A melting curve was performed at the end of the reaction to confirm a single product. Standard curves were performed for each primer set and efficiencies calculated. The data were normalized to glucose-6-phosphate dehydrogenase cDNA expression included with each experiment.

### Gene Ontology Annotation

The predicted protein sequences of H99 serotype A from the Broad assembly 2 (http://www.broadinstitute.org/annotation/genome/cryptococcus_neoformans/Info.html) were submitted to the GOAnno program of AgBase [36]. Each protein sequence was searched against the UniProt database, restricted to fungal sequences. The e-value cutoff for a significant hit was 10^−10^. The sequences and annotations from the Uniprot database were filtered to remove sequences that had only annotations with an evidence code of IEA (Inferred from Electronic Annotation) or ND (No biological Data available). Additional annotation was obtained by submitting those proteins without a match in the UniProt database to InterProScan [37]. Gene ontology assignments were made for domains with matches that had a e-value of <10^−20^. Gene ontology term enrichment was carried out using a hypergeometic test as implemented in the GOstats program, using a p-value of ≤0.01 as a criteria for significance [38].

### Ubiquitination Analysis

*C. neoformans* cells grown in YNB, pH 4.0 were treated with varying concentrations of H_2_O_2_ (0–4 mM) for 30 min. At the end of the incubation, cells were rapidly chilled and collected by centrifugation. Cell pellet was lysed in 8 M urea containing buffer and equal amounts of protein per sample were used for immune blot analysis using antibodies specific to ubiquitin proteins (Rabbit polyclonal to ubiquitin cat # ab19247 from abcam at 4 K dilution). Uniform transfer of proteins across the membrane was verified by staining the membrane with Swift Membrane Stain (cat # 786-677, G BioSciences, MO, USA) before subjecting them to immuno blot analysis.

## Results

### Cells Grown in YNB Medium Exhibited Reduced Capacity to Breakdown Exogenous H_2_O_2_

**Figure 1:**
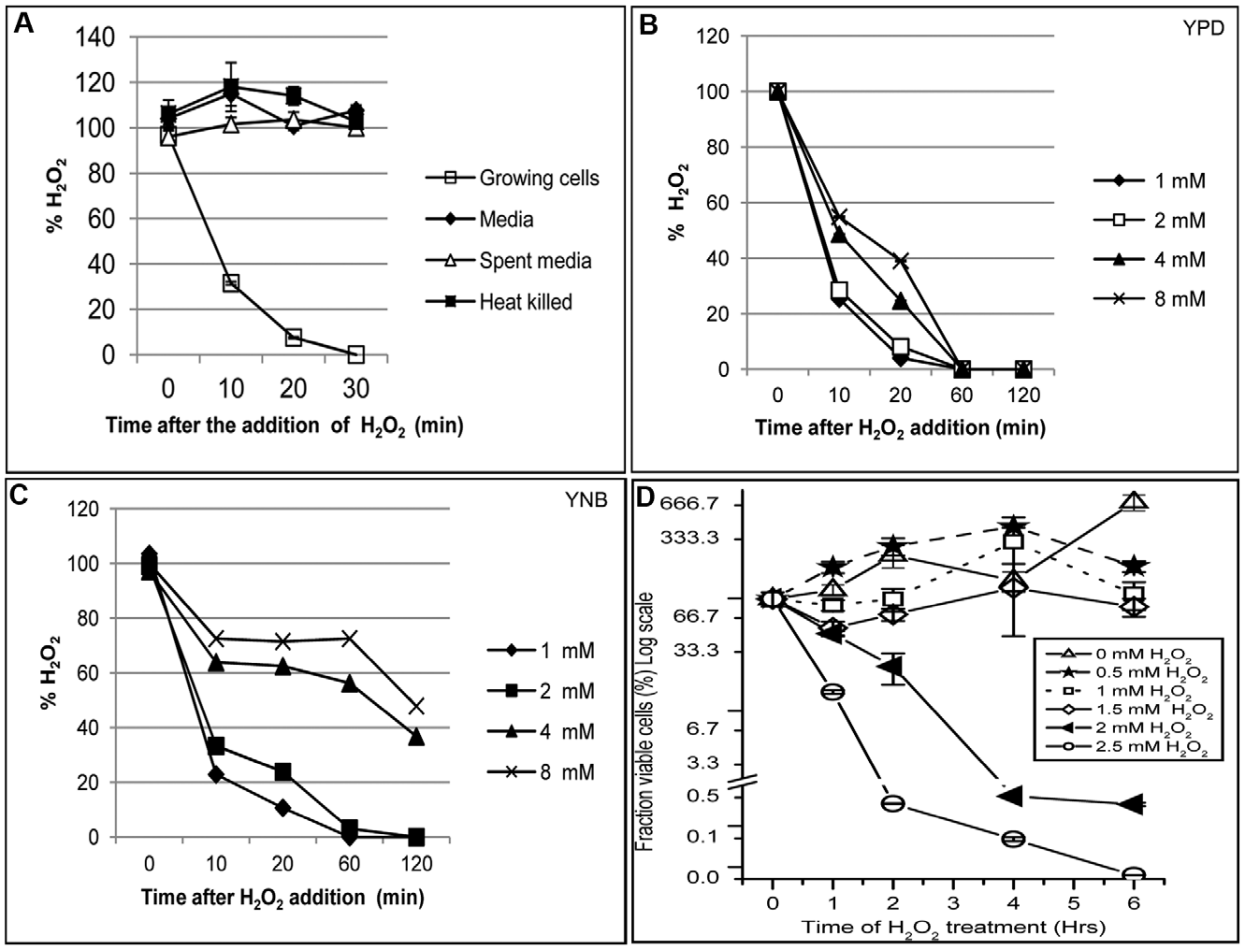
Kinetics of H_2_O_2_ degradation by actively growing *C. neoformans* cells and the effect of concentration of peroxide on fungal viability. **A**: 1 mM H_2_O_2_ was added to KN99 cells growing in YNB, pH 4 at OD_650_ = 1.5 (□), YNB medium alone ( ), spent media (Δ) and to heat killed *C. neoformans* cells (▪). Various concentrations of H_2_O_2_ were added to the cells growing either in YPD (**B**) or in YNB (**C**) medium. At various time points samples were withdrawn, cells were separated by centrifugation and the supernatant was used for the quantitative estimation of H_2_O_2_. Percentage of residual H_2_O_2_ was plotted against treatment time. Error bars reflect standard error calculated from three independent experiments. **D**: Exponentially growing cells at OD_650_ = 1.5 were incubated with various concentrations of H_2_O_2_. At various time points aliquots were withdrawn, cells collected by centrifugation at 4°C, washed two times with cold PBS. Washed cells were serially diluted and plated on solid YPD media, then incubated at 30°C. After 3 days colony forming units (CFU) were counted. Fraction of viable cells was plotted against H_2_O_2_ treatment time.

To understand the fate of exogenous H_2_O_2_ once it has been added to *C. neoformans* culture, actively growing yeast cells in YNB, pH 4 medium were treated with 1 mM H_2_O_2_. At different time intervals aliquot of the cell culture was removed, cells were separated and the supernatant was used to measure the residual H_2_O_2_ in the medium. A concentration of 1 mM H_2_O_2_ was rapidly degraded by growing *C. neoformans* cells and was completely absent in the medium after 30 minutes (Figure 1A). The incubation of H_2_O_2_ either in the medium alone or in the spent medium did not cause significant H_2_O_2_ breakdown over time (Figure 1A). Incubation of heat killed *C. neoformans* cells did not affect the concentration of H_2_O_2_ over time clearly suggesting that actively growing cells are responsible for degradation of external H_2_O_2_.

*C. neoformans* cells are routinely grown in a nutrient rich YPD medium or a nutrient defined YNB medium for laboratory experiments. To investigate the influence of growth media on the capacity of the cells to breakdown exogenous H_2_O_2,_ we treated *C. neoformans* cells either grown in YPD; a nutrient rich medium with a pH of 6.2–6.5 or YNB; a nutrient limited defined medium buffered to pH 4.0, at 30°C with various concentrations of H_2_O_2_. The kinetics of H_2_O_2_ breakdown was followed by measuring the residual H_2_O_2_ in the medium. When the cells were grown and treated in YPD, a concentration of H_2_O_2_ up to 8 mM was completely degraded in one hour at 30°C (Figure 1B). However, the cells grown and treated in YNB, pH 4.0, showed decreased capacity to breakdown exogenous H_2_O_2_, taking up to two hours to completely degrade even 2 mM of H_2_O_2_ (Figure 1C). Higher concentrations of H_2_O_2_ (4 and 8 mM) were decreased to ∼40–50% of the initial concentration by two hours, clearly indicating that unlike the cells grown in YPD, cells grown in nutrient limited YNB medium are much less efficient in degrading exogenous H_2_O_2_.

When the cells were grown to different densities (OD_650_ = 1–8) in either YPD or YNB medium and treated with various concentrations of H_2_O_2_ (2–8 mM), the rate of H_2_O_2_ breakdown increased as the culture density increased (data not shown). A cell culture grown in YPD to a density of OD_650_ = 2.7 was able to degrade 4 mM of H_2_O_2_ in just 20 min, while those with a density of OD_650_ ≥ 4.0 took only 10 minutes (Figure S1A). On the other hand cells grown in YNB, to a density of OD_650_ = 4.0 were unable to completely degrade 4 mM H_2_O_2_ in 2 hrs (Figure S1B). However, cell culture in YNB at a density of OD_650_ = 7.0 completely degraded 4 mM H_2_O_2_ in 10 minutes. Together these data indicate that capacity to degrade exogenous H_2_O_2_ depends not only on the medium but also on the density of the growing culture.

Because *C. neoformans* encounters oxidative stress in a nutrient limited environment inside the phagosome, we chose to use YNB, at pH 4.0 for all of our experiments. We were interested in inducing only oxidative stress response and supplemented YNB medium with 2% glucose and grew the cells at 30°C to prevent the non-specific induction of a transcriptional response due to either carbon source starvation or heat shock respectively. To determine the effect of exogenous H_2_O_2_ on the viability of *C. neoformans* cells, yeast cells grown in YNB, pH 4.0 at an OD_650_ of 1.0 were treated with various concentrations of H_2_O_2_. At various time points samples were withdrawn, cells collected by centrifugation, washed with cold PBS and serially diluted and plated onto YPD plates to count the colony forming units (CFU). A concentration of 0.5 mM H_2_O_2_ had no significant effect on cell viability at any time point (Figure 1D). Treatment of the cells with 1 mM H_2_O_2_ for one hour caused 15% cell death, but by two hours total number of viable cells had recovered to 100% of the initial cell population. Similarly, 1.5 mM H_2_O_2_ caused killing of ∼50% of cells by one hour, however by two hours, the fraction of viable cells increased to 70% of the cell population and by the four hour time point total viable cell population had reached 100%. This suggested that by two hours after addition, the majority of H_2_O_2_ had been degraded and the cell population had begun to recover from the H_2_O_2_ induced damage. Treatment of cells with 2 or 2.5 mM H_2_O_2_ caused a marked decrease in cell viability (Figure 1D) and prolonged incubation in the same medium did not increase their ability to degrade H_2_O_2_ and/or recover. From these results we concluded that sampling 1 mM H_2_O_2_ treated cells across a one-hour time-course should provide a detailed transcriptional response profile of this yeast specific to H_2_O_2_ induced oxidative stress.

### *C. neoformans* Exhibited a Robust Transcriptional Response to Exogenous H_2_O_2_ Treatment

To analyze the transcriptional response of *C. neoformans* to H_2_O_2_, KN99α cells were treated with 1 mM H_2_O_2_ and samples were collected at 5, 15, 30, 45 and 60 minutes. Custom *C. neoformans* serotype A whole genome microarrays were used to measure the differential transcriptional response of cryptococcal cells to perturbation by 1 mM H_2_O_2_ relative to untreated cells. Statistical assessment of differential expression was obtained by fitting the balanced treated and untreated signal intensities to an analysis of variance (ANOVA) model as previously described [39]. We identified 2,930 differentially expressed (DE) probes (P<0.01) at one or more time points representing approximately 45% of the transcriptome represented on the array, indicating a very robust transcriptional response to H_2_O_2_ in *C. neoformans* (Tables 1 and S1).

**Table 1:** Differentially expressed probes at various time points during H_2_O_2_ treatment.

	5 min	15 min	30 min	45 min	60 min
Total (Differentially expressed)	605	1272	2521	1289	835
Up regulated	264	661	1009	592	382
Down regulated	341	611	1512	697	453

**Figure 2:**
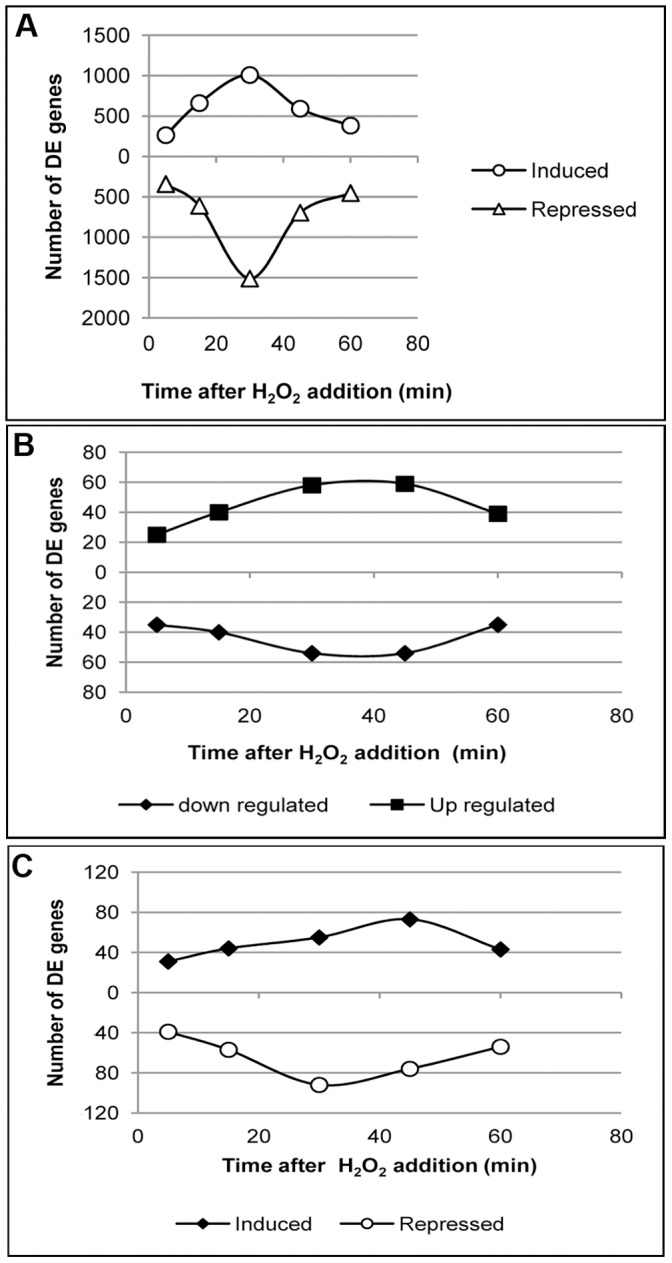
Treatment of *C. neoformans* cells with 1 mM H_2_O_2_ elicited a robust transient transcriptional response. **A:**The number of differentially expressed (DE) genes (up-regulated above X axis and down regulated below X axis) were plotted at various time points during exposure to exogenous H_2_O_2_. **B**: Expression response of *C. neoformans* oxidation and reduction category to H_2_O_2_ treatment. **C**: Transcriptional pattern of differentially expressed genes belonging to metabolic process category during H_2_O_2_ stress.

We measured the transcript changes across a one-hour time-course and identified 605 probes differentially expressed (264 up-regulated, 341down-regulated) at the five minute time point (Table 1). At 15 minutes post-treatment, transcriptional activity increased to a total of 1272 differentially expressed probes (661 up- and 611 down-regulated). Differential expression peaked at the 30-minute sampling, to 2521 probes (1009 up- and 1512 down-regulated). Finally, at 45 and 60 minutes the number of differentially expressed probes decreased to 1289 (592 up- and 697 down-regulated) and 835 probes (382 up- and 453 down-regulated) respectively. Most extensive changes in gene expression were observed between 15 and 45 min after the initiation of the oxidative stress with a peak transcriptional response at 30 min post H_2_O_2_ treatment. The kinetics of transcriptional response (Figure 2A) is consistent with rate of H_2_O_2_ removal from the medium by actively growing yeast cells (Figure 1C).

We compared the sets of genes differentially expressed in response to H_2_O_2_ treatment in our experiment (treatment in YNB medium) to a previously published microarray dataset by Ko et al in which authors employed YPD medium for growth and treatment with H_2_O_2_. Consistent with our present findings on the influence of media composition and treatment conditions on the oxidative stress response we found considerable differences in the gene sets, but a high level of concordance for those genes identified as differentially expressed in both datasets [22]. We compared the identity of the DE transcripts at 30 and 60 minutes with the same two time points in KO et al., data. We observed 1582 DE transcripts compared to the 886 identified by Ko et al. There were 336 transcripts exhibiting overlap at the 30-minute time point on both arrays, with 306 (91%) of those showing the same direction in expression difference. At the 60-minute time point, we observed 836 differentially expressed transcripts compared to 1591 differentially expressed transcripts by Ko et al., with 333 genes overlapping and 276 (83%) of those showing the same direction in expression difference. The low percentage of probes that overlap at the same time point most likely reflects a combination of the differences in media conditions, the kinetics of H_2_O_2_ degradation, array probe differences, strains used and the intrinsic variation of experiments done in two different laboratories. We believe the high degree of concordance between the overlapping probes strongly validates both experimental approaches.

### Quantitative Real Time PCR Experiments Confirmed the Microarray Dataset

Microarray results were validated by performing qRT-PCR analyses of six genes (CNAG_01211, CNAG_02132, CNAG_02288, CNAG_03238, CNAG_04001 and CNAG_06758) that encompassed a range of expression levels at 30 min time point. The mRNA abundance of these transcripts at 30 min after H_2_O_2_ treatment followed similar profile to microarray dataset validating the quality of our array. The linear regression analysis of the microarray and qRT-PCR measurements resulted in a correlation coefficient (*R* value) of 0.94, suggesting that array dataset correlated positively and closely with qRT quantification (data not shown).

### A Large Number of Diverse Biological Processes are Enriched in the Transcriptional Response

Gene ontology (GO) enrichment analysis was performed and several biological processes were significantly enriched in our lists at all time points (Tables S2 and S3). As the number of differentially expressed genes increased at 30 and 45 minute time points (Figure 2A) so did the number of biological processes indicating that differentially expressed genes belong to diverse biological processes. A large number of biological processes perturbed at 30 and 45 minutes post H_2_O_2_ treatment and recovery of the viable cells at two hour time point (Figure 1D) emphasizes the potential importance and combined efforts of these biological processes in combating the H_2_O_2_ induced oxidative stress. The many GO process categories significantly affected by H_2_O_2_ induced stress at all time points include transport, metabolic process, oxidation-reduction, transmembrane transport, cellular response to stress, response to drug, transcription: DNA-dependent, ribosome biogenesis and assembly, pathogenesis, amino acid biosynthetic process, phosphorylation, protein transport, translation, filamentous growth, electron transport chain, signal transduction, cell cycle, ion transport, carbohydrate metabolic process, protein amino acid phosphorylation, fungal-type cell wall organization and biogenesis, lipid biosynthetic process, cell division and mRNA processing.

In addition to the above biological processes, a greater number of unique cellular processes significantly affected only at 30 and 45 min time points were identified (Table S2). They include response to DNA damage stimulus, proteosomal ubiquitin-dependent protein catabolic process, hyphal growth, DNA repair, RNA splicing, ER to Golgi vesicle-mediated transport, ion transport, protein folding, meiosis, chromatin modification, nuclear mRNA splicing via spliceosome, mitosis, tRNA processing, endocytosis, protein amino acid dephosphorylation, electron transport chain, small GTPase mediated signal transduction, vacuolar acidification, DNA replication, GTP catabolic process, carbohydrate metabolic process, sterol biosynthetic process, regulation of translation, and ribosomal large subunit assembly and maintenance. Identification of above GO terms clearly suggests that H_2_O_2_ causes substantial perturbation of cellular processes.

### Oxidation and Reduction Processes are Over-represented in the Transcriptional Response

As expected, our dataset identified oxidation-reduction process as one of the top ten enriched categories affected by H_2_O_2_ treatment (Table S3). Moreover, the number of genes belonging to oxidation and reduction category undergoing DE increased from 60 at the 5-minute time point to 80 at the 15-minute time point. At 30 and 45 minutes post-treatment, there were 112 genes with altered RNA levels that decreased to 74 at the 60-minute time point (Figure. 2B). Some of the genes identified in this category involve those that have previously been demonstrated to be important for oxidative stress resistance either in *C. neoformans* or in other fungal species. These include *TSA*1 coding for thiol specific antioxidase (CNAG_03482) [6], *TRR*1 coding for thioredoxin reductase (CNAG_05847) [15], cytochrome C peroxidase (*CCP1*; CNAG_01138) [19] and catalase genes *CAT*1 (CNAG_0498) and *CAT*3 (CNAG_00575) [40]. These genes exhibited consistent up-regulation at more than one time point (Table S4), suggesting their prolonged involvement in combating the H_2_O_2_-induced stress and further validating the quality of our array.

A more extensive analysis of the genes belonging to the oxidation-reduction functional category revealed potential novel mechanisms of oxidative stress resistance. Functional annotation of the *C. neoformans* genome using our GO database assigned a total of 514 genes to the oxidation-reduction functional category. We discovered that 205 genes exhibited differential expression at one or more time point with a maximum of 124 genes showing altered mRNA levels at 45 min post H_2_O_2_ treatment (Table S4). Reciprocal BLAST search analysis against the *S. cerevisiae* protein database identified potential orthologs for 145 genes (Table S4). Notably, 25 genes showed consistent up-regulation in at least three time points and 25 genes exhibited consistent down-regulation at a minimum of three time points as shown in Table S5. Sustained induction or repression of a gene may reflect its predominant role in oxidative stress resistance. In support of this hypothesis, genes such as *CAT*1, *CAT*3 and *TRR*1, known to be important for oxidative stress either in *C. neoformans* or in other fungal species were found to be up-regulated at three or more time points. The *C. neoformans* homologs of the *S. cerevisiae* genes *ALD*5, *ZTA*1, *SCS*7 and *CIR*2 were also induced at a minimum of three time points (Table S5) and deletion of these genes in *S. cerevisiae* has demonstrated their increased sensitivity to oxidative stress [41], [42], [43].

In addition to the core antioxidant response, our microarray data revealed the contribution of several additional gene products in oxidative stress resistance that may provide clues to novel mechanisms of stress resistance. The strong transcriptional regulation of 50 genes for a significant period of time during H_2_O_2_ treatment suggests an important role for these genes during oxidative stress (Table S4). For example the gene represented by CNAG_00654 was up-regulated at all time points after H_2_O_2_ treatment and is highly similar to sulfiredoxins (*SRX*1). The sulfiredoxins are critical for oxidative stress resistance in yeast and higher eukaryotes [44], [45]. The majority of the remaining gene products have not been characterized either in *C. neoformans* or in other fungal species. A preliminary bioinformatic analysis of a few select genes supported their potential roles during oxidative stress in *C. neoformans* and these are discussed below.

One of the genes that are under persistent transcriptional regulation in response to peroxide stress is CNAG_03238, predicted to belong to dioxygenase subfamily. It is similar to a putative dioxygenase gene from a saprophytic soil borne filamentous bacteria, and shows significant repression at four of the five time points post H_2_O_2_ treatment. Bacterial dioxygenases are reported to have either iron-sulphur center or non-heme mononuclear iron as cofactors. Importantly, these enzymes catalyze oxidation of the substrates at the expense of reduced NADPH [46]. Decreased expression of this gene in *C. neoformans* after H_2_O_2_ treatment suggests that this may be a part of protective mechanism of the yeast cells to inhibit NADPH-requiring reactions while cells are coping with oxidative stress.

Another uncharacterized gene, CNAG_01542, is predicted to have domains belonging to taurine catabolism dioxygenase super-family and exhibits increased expression at 30, 45 and 60 minutes. Proteins containing these domains have been demonstrated to be important for sulfonate metabolism, in the synthesis of Fe-S cluster protein family members and their expression in *S. cerevisiae* provides increased resistance to menadione-induced stress [47]. A gene represented by CNAG_02580, with a conserved 2OG- Fe (II) oxygenase superfamily domain also shows increased expression at 15, 30 and 60 minutes. The 2OG- Fe (II) oxygenase domains are found in enzymes belonging to prolyl hydroxylase (PHD) family and these enzymes play a role in hypoxia and oxidative stress response in *Arabidopsis* and other higher eukaryotes [48]. These preliminary results suggest that systematic characterization of the genes identified in our experiment may reveal the presence of novel mechanisms of oxidative stress resistance.

### *C. neoformans* Elicits a Distinct Pattern of Transcriptional Regulation of Metabolic Process Genes during Oxidative Stress

Among the highly perturbed biological processes, we detected expression changes in numerous genes assigned to the metabolic process category. There are 608 genes assigned to the metabolic process category in the *C. neoformans* genome, with 272 (∼45%) showing differential expression at one or more time points during H_2_O_2_ treatment (Table S6). Of the differentially expressed genes, 98 were common with oxidation-reduction process. The extent of metabolic process perturbation by H_2_O_2_ treatment varied at different time points during the treatment. There were 70 genes exhibiting differential expression at 5 minutes, out of which 31 showed induction and 39 exhibited repression of transcription. The number of differentially expressed genes increased to 101 at 15 minutes with 57 showing repression (Figure 2C). The negative regulation of genes related to metabolic process increased at 30 minutes with 92 genes showing decreased levels of transcripts. However, samples at the 45-minute time point exhibited a significant increase in the number of up-regulated genes, suggesting that as the concentration of H_2_O_2_ in the medium decreases, there is a shift in the metabolic flux and reprogramming of metabolic processes. Interestingly even at the 60-minute (30 minutes after the complete absence of H_2_O_2_ from the medium) a majority of the metabolic process related genes were still under transcriptional regulation (Figure 2C). Out of the 272 metabolic process related genes displaying altered transcription, we detected 33 genes exhibiting consistent up-regulation at three or more time points and 45 genes had their transcript level decreased at three or more time points after H_2_O_2_ treatment (Table S6).

A subset of genes showing distinct differential expression pattern under metabolic process category are homologous to genes involved in ergosterol metabolism and antifungal drug resistance in other fungal species. Transient differential expression of a particular set of genes (related to ergosterol metabolism and antifungal drug resistance has been reported to contribute to the complex mechanisms of anti-fungal drug resistance [49]. Over expression of *ERG11* has been reported in clinical isolates of drug resistant *C. albicans,* implicating its role in fungal drug resistance [50]. We observed persistent up-regulation of one of the identified homologs of *S. cerevisiae ERG11* after H_2_O_2_ treatment (CNAG_ 05842, *ERG110,* and Figure S2). In *S. cerevisiae ERG5* has been reported to bind to azole drugs by a similar mechanism as *ERG11* and we observed increased expression of *C. neoformans ERG5* (CNAG_06644) as well. Sterol analysis of the flucanazole resistant *C. albicans* showed defects in *ERG2* and *ERG3* [51]. Homologs of *S. cerevisiae ERG2* (CNAG_00854) and *ERG3* (CNAG_00519) showed altered expression levels in our microarray dataset (Figure S2). In addition to the above, we have observed altered regulation of 12 *C. neoformans* genes upon peroxide stress that are homologs of *S. cerevisiae* ergosterol biosynthesis genes (Figure S2) clearly suggesting a cross-talk between the mechanisms of oxidative stress and anti fungal drug resistance. Several families of ABC transporters have been characterized as efflux pumps in *S. cerevisiae* and *C. albicans* and are important for drug resistance [52]. The over expression of genes encoding two of the *PDR5* family transporters in *C. albicans CDR1* and *CDR2* as well as that of a major facilitator Ca*MDR1* is one of the signature characteristics of resistant isolates [49], [53]. The *C. neoformans* genome contains two homologs of *CDR1* (CNAG_04098, CNAG_04966) and a homolog of *MDR1* (CNAG_00796). All three genes show differential transcriptional regulation, with *CDR11* showing consistent up regulation at 15, 30 and 45 minutes and *MDR1* exhibiting increased levels at 30, 45 and 60 minutes (Figure S2).

### Cytochrome C Peroxidase Mediated Respiratory Chain is Essential for Oxidative Stress Resistance

**Figure 3:**
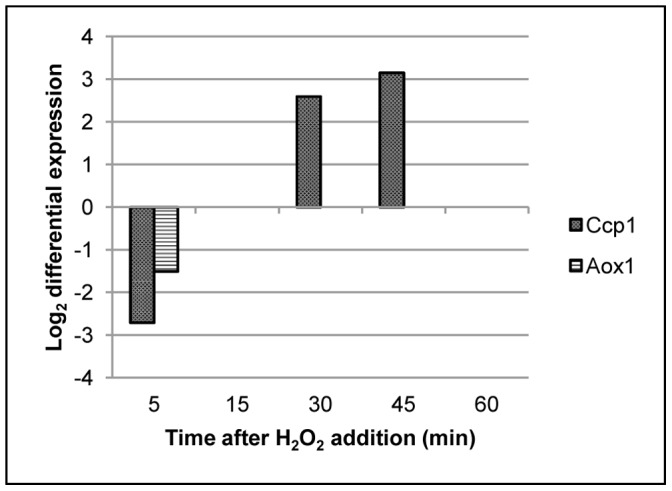
Transcript abundance of *C. neoformans CCP1* and *AOX1* at various time points during H_2_O_2_ treatment.

The mitochondrion is the site of most oxidation-reduction processes and we were particularly interested in the differential expression pattern of *CCP*1, which codes for cytochrome C peroxidase (CNAG_01138), and *AOX1,* encoding an alternative oxidase (CNAG_00162). *C. neoformans CCP*1 is known to protect against external oxidative stress inducing agents and consistent with that role, its transcription was increased at 30 and 45 minutes (Figure 3 and [19]). *C. neoformans AOX1* is important for fungal pathogenesis and this phenotype has been attributed to its role in oxidative stress resistance [20]. However, transcription of *AOX1* gene was not altered in our array after H_2_O_2_ treatment in the same manner as *CCP1* (Figure 3). H_2_O_2_ treatment caused an initial repression of *AOX*1 mRNA levels with no further changes in its expression at subsequent time points. Therefore we decided to explore the potential functional consequences of the differential expression of these two genes in response to H_2_O_2_ induced oxidative stress.

**Figure 4:**
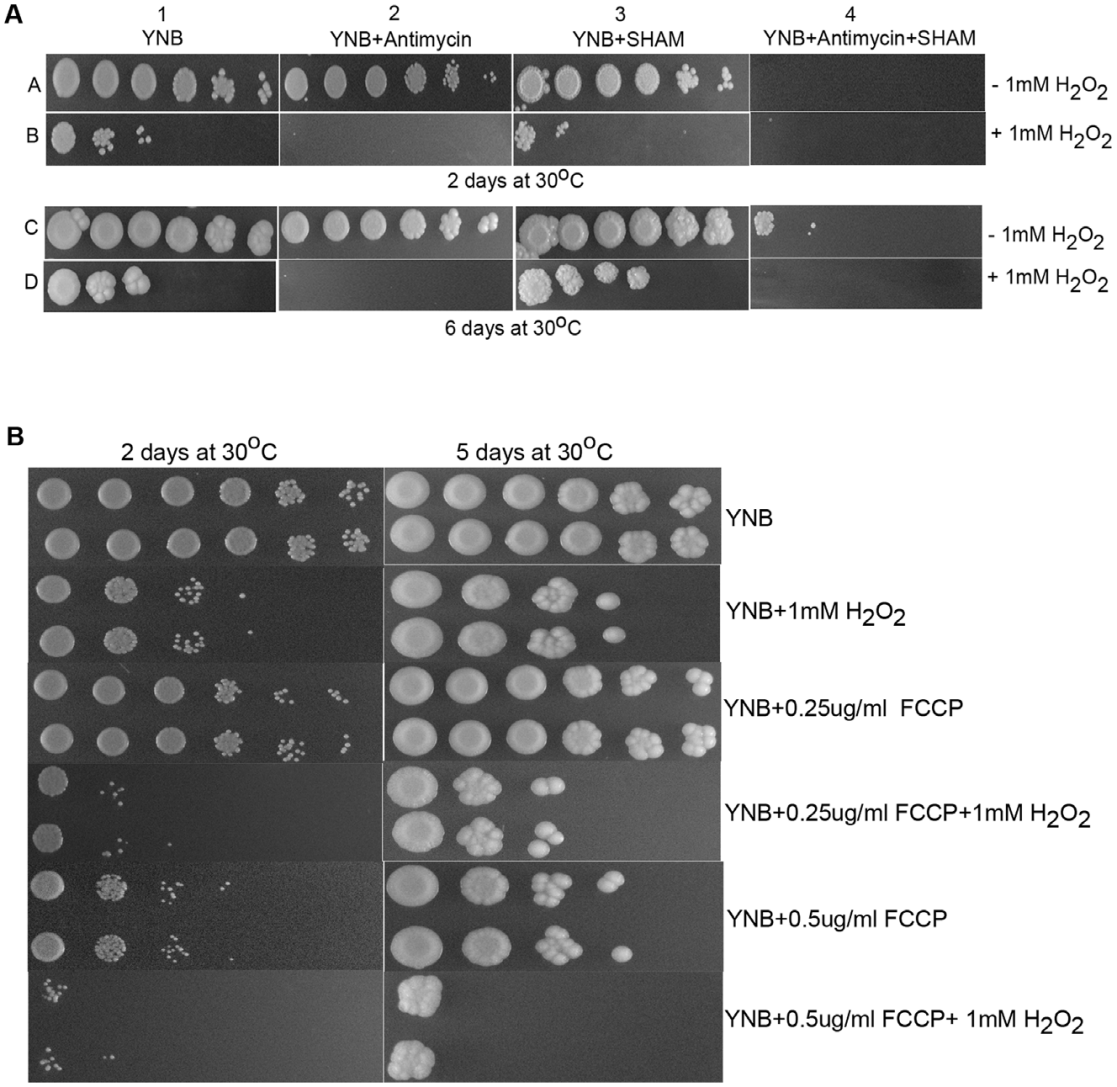
The affect of inhibitors of electron transport chain and mitochondrial function on C. *neoformans* oxidative stress resistance. **A**: Logarithmically growing KN99 cells in YNB were 10 fold serially diluted and 5 ul of the cell suspension was spotted onto YNB agar containing either 0.5 ug/ml of antimycin (2) or 2 mM SHAM (3) or mixture of antimycin and SHAM (4) either in the absence (A and C) or in the presence (B and D) of 1 mM H_2_O_2_.**B**: Two independent cultures of *C. neoformans* cells were grown in YNB. At OD_650_ = 1.5, cells were collected, 10 fold serially diluted and 5 ul of the cell suspension was spotted onto solid YNB agar plates containing either 0.25 ug/ml or 0.5 ug/ml of FCCP in the presence or absence of 1 mM H_2_O_2_. Plates were incubated at 30°C for various days and photographed.

During mitochondrial respiration, *C. neoformans* utilizes both the classical electron transport chain, mediated by *CCP*1and an alternative oxidase chain mediated by *AOX*1 for the synthesis of ATP. These two pathways have been demonstrated to be functionally redundant in *C. neoformans* using specific respiratory chain inhibitors [20]. To elucidate the role of these two electron transport chains during H_2_O_2_ treatment, we treated cells with specific mitochondrial pathway inhibitors in conjunction with H_2_O_2_. The growth of KN99 cells in the presence of either antimycin, an inhibitor of the cytochrome c peroxidase pathway, or SHAM, an inhibitor of the alternate oxidase pathway, was found to be similar to that of the control plate (Figure 4A, A1–A3 and C1–C3). However, addition of both antimycin and SHAM resulted in severe impairment of growth of *C. neoformans* cells (Figure 4A, A4 and C4). Treatment of the cells with H_2_O_2_ in the presence of antimycin caused complete loss of growth when observed at 2 days (Figure 4A, B2) or at 6 days at 30°C (Figure 4A, D2). When cells were subjected to H_2_O_2_ stress in the presence of SHAM alone, slight growth was observed after two days (Figure 4A, B3) however, significant growth was restored after 6 days of incubation (Figure 4A, compare D1 to D3). Exposure of the cells to H_2_O_2_ in the presence of both antimycin and SHAM resulted in their complete loss of resistance to exogenous H_2_O_2_ (Figure 4A, compare panels B1 with B4 and D1 with D4). This provides evidence for a major role of *CCP1* during H_2_O_2_ induced stress with a more limited role for the Aox1 protein, consistent with their differential expression pattern (Figure 3).

The preferential role of *CCP*1 during H_2_O_2_ induced oxidative stress was intriguing since the flow of electrons through the *CCP1* mediated pathway generates more ROS than respiration through the *AOX1* pathway [54]. It is plausible that utilization of the *AOX1* mediated pathway during H_2_O_2_ induced stress may provide an additional benefit by minimizing the release of internal ROS. However, a potential advantage of using the *CCP1* mediated pathway is its higher efficiency of ATP synthesis due to more proton pumping sites along the path of electron flow compared to the *AOX1* mediated route. Therefore, we hypothesized that *C. neoformans* preferentially utilizes the *CCP1* mediated electron transport chain to generate more ATP with which to carry out energy dependent processes including the repair of damage caused by H_2_O_2_ induced oxidative stress.

### Functional Mitochondria are Essential for Protection Against Oxidative Stress

Additional roles for mitochondrial function during H_2_O_2_-induced oxidative stress were revealed by further analysis of the differential expression pattern of genes related to oxidation-reduction process. We identified 50 genes whose expression was either induced or repressed at a minimum of three time points during H_2_O_2_ (Table S5). Of the 50 oxidation-reduction genes altered at a minimum of three time points, 39 had potential homologs in *S. cerevisiae*. Out of those 39 genes, 22 are known or predicted to be located in the mitochondria (Table S5) according to their annotation in the *Saccharomyce*s genome database [55]. The involvement of *C. neoformans* mitochondria during *in vitro* oxidative stress or while they are being phagocytosed is not well understood, though functional mitochondria are indispensable for growth inside the host [56]. The global transcriptome profile of *C. neoformans* at the site of central nervous system infection revealed higher expression of several respiratory genes demonstrating the importance of mitochondrial function for growth inside the host [57], [58]. Most fungal pathogens exhibit a distinct arrangement of electron transport chain compared to their host and a more flexible and complex pathways of electron flow that have not yet been fully elucidated [59]. The recent characterization of the hyper-virulent *C. gatti* responsible for the recent fatal fungal outbreak in Vancouver Island provides evidence for the potential role of mitochondria during oxidative stress [60]. In these studies the authors attributed the hyper-virulence to an increased intercellular proliferation rate of the fungal cells. In a phenotypic screen to identify fungal factors responsible for enhanced intracellular proliferation rate inside the host macrophage cell lines, Ma et al, identified a unique tubular mitochondrial morphology of yeast cells that positively correlated with enhanced intracellular parasitism, demonstrating a strong link between mitochondrial regulation and growth inside the macrophages [60]. Therefore we decided to explore the role of mitochondrial function in H_2_O_2_ induced oxidative stress.

To test the importance of mitochondrial function during H_2_O_2_ induced stress, we treated cells with a protonophore FCCP, known to disrupt mitochondrial function in other fungal organisms [61], [62]. *C. neoformans* cells were very sensitive to FCCP, with concentrations as low as 0.5 ug/ml affecting their growth in liquid culture (data not shown). By contrast, yeasts such as *C. albicans* and *S. cerevisiae* cells were able to grow normally in the presence of 2.5–5 ug/ml of FCCP [61], [62]. Because of this sensitivity, we used concentrations of 0.25 and 0.5 ug/ml of FCCP for our studies. Two independent cultures of KN99 cells were spotted on plates containing 1 mM H_2_O_2_ in the presence and absence of FCCP and incubated at 30°C. The capacity to withstand oxidative stress triggered by H_2_O_2_ challenge was markedly decreased by FCCP in a concentration dependent manner (Figure 4B), providing further support for the important role of functional mitochondria in oxidative stress resistance in *C. neoformans.*

### Ubiquitin-dependent Protein Catabolic Genes are Sensitive to Oxidative Stress

The ubiquitin dependent proteosome pathway is one of the several cellular processes requiring ATP and is critical for maintaining cellular homeostasis. Protein ubiquitination is important during various stress conditions in yeast and higher eukaryotes and, more recently, protein ubiquitination has been shown to be important for stress resistance, adaptation and virulence of the human fungal pathogen *C. albicans* [63]. Moreover a previous microarray study on the oxidative stress resistance of *C. neoformans* found that a *ubc*8 deletion strain is more sensitive to H_2_O_2_ *in vitro*, demonstrating the importance of ubiquitin related processes for oxidative stress resistance [22].

**Figure 5:**
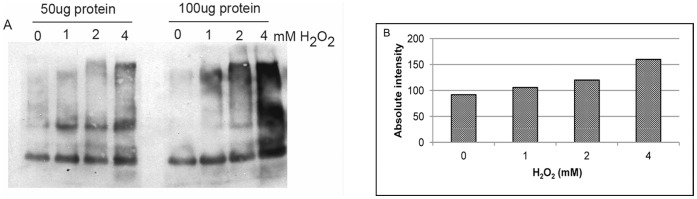
H_2_O_2_ induced oxidative stress stimulates increased ubiquitination of *C. neoformans* proteins. (**A**): Immuno blot analysis of the proteins from *C. neoformans* cells growing in YNB, pH 4 at OD_650_ = 1.5 and treated with various concentrations (0, 1, 2 and 4 mM) of H_2_O_2_. Cell lysates were prepared in urea containing denaturing buffer. Equal amount of protein was electrophoresed on SDS-polyacrylamide gel, transferred to nitrocellulose and ubiquitin conjugates were probed with polyclonal anti-ubiquitin antiserum. (**B**): Densitometric quantification of the signal from immunoblot A.

We identified 186 genes that were assigned the ubiquitin related biological process terms protein polyubiquitinylation (GO: 0000209), deubiquitinating enzyme (GO: 0004843), ubiquitin-dependent protein catabolism (GO: 0006511), protein ubiquitinylation (GO: 0016567), and proteosomal ubiquitin-dependent protein catabolism (GO: 0043161) in the *C. neoformans* genome (Table S7). Of the 186 genes, 91 (49%) exhibited significant differential expression at one or more time points during H_2_O_2_ treatment (Table S7). In *S. cerevisiae,* proteins encoded by the ubiquitin conjugating (*UBC*) gene family have been implicated in a wide variety of cellular functions including those closely connected with oxidative stress resistance such as respiratory growth, glutathione homeostasis, UV and metal ion resistance and double strand DNA repair [64], [65]. Accordingly individual *UBC* gene deletion strains exhibit varying degree of sensitivities to diverse external stress conditions indicating the functional overlap between different UBC gene products [64], [66]. As it is likely that single ubiquitin related gene products are involved in multiple cellular stress conditions, we employed a biochemical strategy to directly address the role of ubiquitination during oxidative stress. One of the affects of H_2_O_2_ induced oxidative stress involves protein damage by irreversible oxidation and these oxidized proteins are recycled through the ubiquitin dependent proteasomal pathway in other systems [67]. To verify whether similar mechanisms operate in *C. neoformans*, we treated fungal cells with increasing concentration of H_2_O_2_ and subjected the cell lysates to immuno-blot analysis. The blots were probed with ubiquitin specific antibody to quantify the amount of ubiquitin tagged proteins in treated cells to their untreated counterpart. We observed an H_2_O_2_ concentration dependent increase in the amount of ubiquitin conjugated proteins in the total cell lysate (Figure 5A). Densitometric analysis of the immuno blot clearly showed direct correlation between the effective concentration of H_2_O_2_ used in the treatment and the number and intensity of ubiquitinated proteins in total cell lysate (Figure 5B).

## Discussion

The transcriptional regulation of protective mechanisms against oxidative stress is critical for *C. neoformans* both for establishing an infection in the host and also for surviving extended periods of time in the environment. The kinetics of H_2_O_2_ degradation by cells growing in YNB indicates that actively growing *C. neoformans* cells have an efficient mechanism to degrade exogenous H_2_O_2_. This may involve the activity of functional catalase enzymes reported to be present in *C. neoformans* [40]. The absence of H_2_O_2_ degrading activity in the culture supernatant is consistent with an earlier study reporting the absence of secreted catalase family proteins in *C. neoformans* genome [40]. The higher capacity of H_2_O_2_ breakdown by cells growing in YPD compared to those grown in YNB was rather surprising and clearly suggests that growth media composition may significantly affects cell’s constitutive redox potential. Moreover, we also demonstrated that cells grown to a higher optical density were able to degrade exogenous H_2_O_2_ more rapidly compared to the culture at lower optical density, again emphasizing that the cell culture and treatment conditions may significantly influence the magnitude and intensity of the induced cellular transcriptional response to oxidative stress. Therefore these important experimental parameters need to be considered when comparing different datasets generated for H_2_O_2_ mediated stress induced either in the same organism or between different organisms.

At every time point, both induced and repressed genes displayed reciprocal transient profiles with negative regulation of transcription more predominant at all 4 of the 5 time points post H_2_O_2_ treatment (Figure 2A and Table 1). It took almost 30 minutes for the cells to exhibit maximum differential expression, even though the vast majority of the H_2_O_2_ is degraded by that time. The amount of time it takes for cells to maximally respond transcriptionally may depend on the growth history of the cells prior to H_2_O_2_ challenge. During the initial stages of encounter with H_2_O_2_ cells may use their preexisting pool of antioxidant defenses to neutralize H_2_O_2_, and the concentration of cellular antioxidant molecules may likely vary depending on the environment in which cells are grown and treated. Accordingly, the duration, intensity and the complexity of the elicited transcriptional response will differ depending on the environment in which *C. neoformans* exist prior to H_2_O_2_ treatment. It is plausible that the antioxidant capacity of the cells in YPD is significantly higher than those grown in YNB and thus cells grown in YPD may require a higher concentration of H_2_O_2_ to exhibit similar magnitude of transcriptional response at 30 min after treatment, compared to those grown in YNB. This observed influence of growth conditions on the sensitivity of yeast cells to peroxide stress may be of special importance in *C. neoformans* pathogenesis since various species of *Cryptococcus* has been discovered to be associated with diverse ecological niches such as avian guano, vegetables, wood, dairy products, and soil [68], [69]. The composition of these environmental conditions may therefore determine the level of yeast cells’ constitutive redox potential. The ability to cause infection in a mammalian host will depend on their capacity to resist initial oxidative burst inside the macrophages. Therefore, increased resistance to oxidative stress as a result of environmental factors may play a role in enhanced virulence during infection.

In the oxidation-reduction category we found interesting gene regulation patterns of thioredoxin family genes. Among the genes of thioredoxin family, we found increased expression of *TSA*1 (CNAG_03482) at the 15 and 30 minute time points consistent with its established role in oxidative stress and virulence [6]. *C. neoformans TSA1* is a typical 2-cys peroxiredoxin and its homolog in *S. cerevisiae* is located in the cytoplasm [70]. *C. neoformans TSA3* (CNAG_06917) and *DOT5* (CNAG_02854) belong to 1-cys peroxidase family and have shown increased expression at both protein and mRNA levels in response to oxidative stress in a previous study [6]. The homolog of *C. neoformans TSA3* and *DOT5* in *S. cerevisiae* are located to mitochondria and nucleus respectively and are known to play a major role in oxidative stress resistance [70]. The sub-cellular location of Tsa3 and Dot5 in *C. neoformans* is not known and their deletion in *C. neoformans* did not affect oxidative stress resistance in either *in vitro* or *in vivo* conditions [6]. The strong negative regulation of both *TSA3* and *DOT5* shown in our dataset is similar to the pattern of expression of the majority of genes of potential mitochondrial origin suggesting that both *TSA3* and *DOT5* may contribute additionally to oxidative stress resistance in *C. neoformans* by unknown mechanisms.

Three of the four known catalase genes in *C. neoformans* showed differential expression. Increased expression of *CAT1* is consistent it being the only functional catalase as determined by the in-gel activity staining of *C. neoformans* cell extracts [40]. The similar profile of *CAT1* and *CAT3* suggests these genes may be regulated by similar factors, consistent with both belonging to the same phylogenetic clade [40]. However, computational analysis of the promoter regions of these two genes in the past has not identified any conserved cis-acting elements [40]. Interestingly both these genes show increased amounts of mRNA even at 60 minutes while by 30 minutes all the H_2_O_2_ in the medium had been degraded. This suggests that the *CAT1* and *CAT3* gene products may have additional novel roles during *in vitro* oxidative stress induced by H_2_O_2_.

In addition to their initial exposure to oxidative stress inside the macrophages, *C. neoformans* undergoes major metabolic adaptation during growth inside the host [57], [71]. The majority of the metabolic changes discovered during infection so far have been attributed to the task of growing in a limited nutrient environment. We observed significant differential expression of genes assigned to the metabolic process functional category, despite the presence of 2% glucose in our study (Table S6). Widespread differential expression of the genes related to metabolic processes was also observed during nitrosative stress in the presence of 2% glucose, suggesting the presence of overlapping mechanisms of oxidative and nitrosative stress protection [72]. A unique feature of the metabolic process genes induced by H_2_O_2_ involves the genes encoding pentose phosphate pathway (PPP) enzymes. A major product of PPP is the production of NADPH that is critical for the function of proteins required for repairing oxidative protein damage. Accordingly the components and function of PPP have been shown to be important for resistance and adaptation to oxidative stress in yeast and higher eukaryotes. In both *S. cerevisiae* and *C. glabrata*, H_2_O_2_ treatment increased the expression of genes belonging to the pentose phosphate pathway [73], [74]. Moreover, expression of glucose 6-phosphate dehydrogenase (*ZWF1*) is increased during exposure of *C. albicans* to nitrosative stress [75]. In *S. cerevisiae*, independent deletion strains of pentose phosphate pathway genes such as 6-phospho gluconate dehydrogenase (*GND1*), D-ribulose-5-phosphate 3-epimerase (*RPE1*), transketolase 1 and transketolase 2 (*TKL1*and *TKL2*), glucose-6 phosphate dehydrogenase (*ZWF1*) and transaldolase (*TAL1*) all exhibit increased sensitivity to oxidative stress [76]. Interestingly, we observed no differential expression of PPP genes, suggesting a more limited role of this metabolic pathway during oxidative stress in *C. neoformans*. Previous studies demonstrated that neither the *ZWF1* mRNA nor protein levels were altered during nitrosative stress in *C. neoformans.* Consistent with this deletion of *ZWF1* gene in *C. neoformans* did not increase their sensitivity to either oxidative or nitrosative stress [72], [77]. These are consistent with our present observations that pentose phosphate pathway does not participate in peroxide induced oxidative stress resistance in *C. neoformans*.

A major mechanism of adaptation to carbon source limitation during growth inside the host is increased expression of genes belonging to carbon metabolism [71]. We observed increased expression of three genes from the TCA cycle, aconitase, succinate dehdrogenase and malic enzyme (Table S6). Increased TCA cycle activity may drive the rate of electron flow through the *CCP1*-mediated mitochondrial electron transport chain to meet the sudden increase in demand for ATP necessary for repairing damaged proteins. Previous serial analysis of gene expression (SAGE) revealed a high abundance of tags corresponding to phopsphophenol pyruvate carboxykinase (*PCK1)*, a main control enzyme for the regulation of gluconeogenesis in the lung-exposed cryptococcal library suggesting that gluconeogenesis is important for fungal survival in the host lung due to the limited availability of glucose [71]. Interestingly, in spite of exposing *C. neoformans* cells to H_2_O_2_ in the presence of glucose we also observed significant and consistent up-regulation of *PCK1* (CNAG_04217) after H_2_O_2_ treatment (Table S6). The genes encoding glycolytic functions including glucose 6-phosphate isomerase (CNAG_03916) and phosphoglycerate mutase (CNAG_05892) showed decreased expression in our dataset similar to their low abundance in the lung-exposed cryptococcal SAGE library [71]. Decreased glycolysis with concomitant increase in gluconeogenesis may be critical for the regeneration of sugar phosphates that are the substrates of nucleotide biosynthesis, glycosylation and cell wall biosynthesis processes. The role of these processes during peroxide stress is supported by their identification after the GO analysis of the differentially expressed genes (Table S3). Further, the importance of *C. neoformans* cell wall biogenesis during oxidative stress through protein kinase C mediated cell integrity pathway has already been documented [78].

Unlike the response of the TCA cycle and the gluconeogenic pathway, genes of the glyoxylate cycle that converts acetyl-CoA to succinate for the synthesis of carbohydrates responded completely differently in our dataset compared to the previously published lung exposed cryptococcal SAGE library [71]. We observed no elevated expression of acetyl-CoA synthase (*ACS1*), malate synthase (*MLS1*), pyruvate decarboxylase (*PDC1*) or alcohol dehydrogenase (*ADH1*) during oxidative stress in contrast to their increased expression during growth conditions in the host lung. The up-regulation of the glyoxylate pathway during pulmonary infection conditions has been attributed to the higher amount of acetate present in lung tissues [71]. This is consistent with the absence of the differential expression of genes related to glyoxylate pathway in our treatment conditions and also confirms the specificity of the differential expression response we obtained to the oxidative stress induced by H_2_O_2_.

Peroxide-induced oxidative stress also caused significant perturbation of genes of amino acid biosynthesis pathway (Table S6). Increased amino acid biosynthetic gene expression has also been reported in *C. neoformans* during nitrosative stress [72]. It is possible that amino acid biosynthesis pathway has important overlapping functions in oxidative and nitrosative stress resistance.

In *S. cerevisiae* transcriptional up-regulation of *CCP1* after H_2_O_2_ treatment and its role in stress signaling has been reported to contribute to oxidative stress resistance [79], [80]. While *C. neoformans CCP1* was initially identified by bioinformatic analysis, the *AOX1*gene was identified by its significant up-regulation in *C. neoformans* in response to exposure to 37°C temperature [19], [20]. However, in a separate study employing SAGE during early murine pulmonary infection studies, authors identified tags for *AOX1* as being enriched in the mouse lung library, but not *CCP1* [71]. These results indicate that transcriptional regulation of *C. neoformans CCP1* and *AOX1*are potentially subject to different mechanisms. Moreover, the *AOX1* deleted strains exhibited a slight virulence phenotype in a mouse model while *CCP1* deleted strains were avirulent. However, both *CCP1* and *AOX1* independent gene deletion strains exhibited *in vitro* oxidative stress sensitivity towards H_2_O_2_ and tert-butyl hydroperoxide respectively [20], [58]. The differential expression pattern of *CCP1* and *AOX*1 observed in our dataset in response to peroxide stress further supports that their transcription is under the control of different signaling mechanisms. The absence of *AOX1* up regulation seen in our dataset is similar to the one reported in the previously published microarray analysis [22]. Moreover transcriptional response of *AOX1*observed in our microarray analysis was in agreement with the results obtained in our laboratory when we subjected RNA samples to RNA seq analysis (unpublished results). The functional consequence of differential expression of *CCP1* during H_2_O_2_ treatment is dramatic and is reflected in the ability of its specific inhibitor antimycin to completely abolish oxidative stress resistance to H_2_O_2_, whereas specific inhibition of *AOX1* had little effect on oxidative stress resistance.

The different arrangement of fungal proteins in the electron transport chain compared to their hosts points to the special importance of mitochondrial function in pathogenesis. This is supported by recent reports showing an active role for functional mitochondria in fungal virulence and drug resistance [81]. The significant perturbation of expression of genes related to mitochondrial function in our microarray is not surprising since earlier gene expression studies in *C. neoformans* in the presence of various stress conditions such as nitrosative stress, heat shock and growth inside the host, all identified genes related to the mitochondria and respiratory chain to be differentially expressed [57], [72]. FCCP has previously been used to investigate mitochondrial function in other yeasts such as *S. cerevisiae* and *C*. *albicans* [62], [82]. The inhibition of ATP synthesis by FCCP and the subsequent increased sensitivity of yeast cells to H_2_O_2_ provides evidence that *C. neoformans* cells undergoes substantial damage by H_2_O_2_ and energy dependent repair mechanisms are critical for recovering from oxidative stress. The increased concentration of ubiquitin tagged proteins observed by us during H_2_O_2_ treatment (Figure 5) further supports the requirement of ATP for damage repair after oxidative stress exposure.

Permanent oxidation of proteins disrupts their structure and debilitates their function. The ubiquitin-dependent proteasomal system is part of the protein degrading mechanism that helps maintain cellular homeostasis. The covalent attachment of ubiquitin to proteins as a selection for degradation is the hallmark of the ubiquitin-dependent pathway. ATP-dependent degradation of ubiquitinated proteins is catalysed by the 26S proteasome complex that is composed of a 20S core particle and a 19S regulatory particle. The observation that exposure of *C. neoformans* to H_2_O_2_ causes increased ubiquitination of cellular proteins (Figure 5) is consistent with the significant induction of *UBI*4 (CNAG_01920). *UBI4* dependent ubiquitination has been shown to play a major role in the ubiquitin dependent protein degradation pathway during thermal, cell wall and oxidative stress conditions in *S. cerevisiae* and *C. albicans* [63], [83]. We observed up-regulation of homologs of *S. cerevisiae RSP5* (CNAG_05355) (Table S7). *RSP5* encodes an essential E3 ubiquitin ligase in *S. cerevisiae*, and its transcription pattern is similar to *UBI4*, indicating that it may be required for ligating potential substrates with ubiquitin.

Deletion mutants of ubiquitin conjugating enzymes *UBC4* and *UBC5* in *S. cerevisiae* were exceedingly sensitive to stress conditions [66]. The increased interaction of Ubc4p with 26S proteasome has been shown in *S. cerevisae* upon heat stress [84]. The homolog of *S. cerevisiae UBC4* in *C. neoformans* (CNAG_01084, Table S7) exhibits persistent expression at four time points, suggesting its major role in recognizing oxidatively damaged substrates. A slight induction of (at only one time point, Table S7) homologs of *S. cerevisiae UBC6* and *UBC8* in *C. neoformans* suggests that they may have a weaker affinity towards the oxidized protein substrates. Proteins encoded by homologs of *S. cerevisiae UBC1*, *UBC2*, *UBC13*, *UBC5*, and *UBC12* all showed decreased expression, further supporting the major role of only Ubc4 enzyme in recognizing and diverting damaged proteins to proteasome pathway for their degradation during oxidative stress. *C. neoformans RAD4, RAD16* and *RAD7* were continuously induced for the majority of H_2_O_2_ treatment time (Table S7). *S. cerevisiae* Rad7p has a functional ubiquitin ligase activity and its interaction with Rad16p and Rad4p are critical for the proteasome dependent nucleotide excision repair [85]. Increased amount of *C. neoformans* Rad4, Rad16 and Rad7 suggests that they may be involved in the proteasomal dependent repair of DNA damage caused by H_2_O_2_ induced oxidative stress. We observed repression of the majority of genes coding for the 19S regulatory particle of the proteasome *RPN1*, *RPN*2, *RPN*5, *RPN*6, *RPN*7, *RPN*8, *RPN*9, *RPN*11 and *RPN12*) and RPT gene (*RPT3*, *RPT*4, *RPT*5 and *RPT*6) families. Several recent reports in yeast (*S. cerevisiae*) and mammalian systems indicate that degradation of oxidized proteins may occur either by ubiquitin/ATP-dependent (catalysed by 26S) or ubiquitin/ATP-independent (catalysed by 20S) mechanisms [67], [86], [87]. Moreover the 20S proteasome complex alone has been shown to possess the capacity to recognize and degrade oxidatively damaged proteins in vitro [67], [86], [87]. This was also supported by observations that oxidative stress stimulates the separation of the 19S regulatory particle from the 26S core particle to yield the 20S proteasome complex [88]. The RPN and RPT family of genes belong to the 19S proteasome complex. The repression of these genes during H_2_O_2_ treatment indicates that the 19S regulatory particle may play a minor role in maintaining cellular homeostasis during oxidative stress in *C. neoformans*.

By examining gene expression differences over a time course that paralleled the kinetics of H_2_O_2_ removal, we identified many more genes affected by oxidative stress than were identified in previous studies of oxidative stress in *C. neoformans*. The pattern of the transcriptional response mirrored the kinetics of peroxide removal and allowed us to infer potential mechanisms for the response as well as the recovery from oxidative stress. We found potential novel mechanisms for the role of mitochondria and expanded our understanding of the role of ubiquitin-dependent proteolysis in recovery from oxidative e stress.

## Supporting Information

Figure S1 **The affect culture density on H_2_O_2_ breakdown by** ***C. neoformans*** **cells.** A 4 mM of H_2_O_2_ was added to cultures at various densities (OD_650_) growing in YPD (A) and YNB (B). At various time points samples were withdrawn, cells separated by centrifugation and the supernatant was used for H_2_O_2_ estimation. The percentage of residual H_2_O_2_ was plotted against H_2_O_2_ treatment time. Standard bars reflect standard error calculated from three independent experiments.
(TIF)

Figure S2 **Differential expression pattern of** ***C. neoformans*** **genes related to anti-fungal drug resistance upon peroxide stress.**
(TIF)

Table S1 **Global transcriptional profile of** ***C. neoformans*** **genome during H_2_O_2_ induced oxidative stress.**
(XLSX)

Table S2 **Gene ontology enrichment analysis of the differentially expressed probes at various time points during H_2_O_2_ treatment.**
(PDF)

Table S3 **Master list of gene ontology annotation of the differentially expressed probes at various time points.**
(XLSX)

Table S4 **Differential expression pattern of** ***C. neoformans*** **genes assigned to oxidation-reduction functional category at various time points during exposure to oxidative stress.**
(XLSX)

Table S5 **List of** ***C. neoformans*** **differentially expressed genes assigned to oxidation-reduction functional category that exhibited persistent induction or repression for a minimum of three time points during H_2_O_2_ induced oxidative stress.**
(PDF)

Table S6 **Differential gene expression profile of the genes belonging to metabolic process functional category with proposed gene names.**
(XLSX)

Table S7 **Transcript abundance at multiple time points of the genes belonging to** ***C. neoformans*** **ubiquitin dependent protein catabolic processes during H_2_O_2_ induced oxidative stress.**
(XLSX)
