# Supplementary figures and images for: Global Transcriptome Profile of Cryptococcus neoformans during Exposure to Hydrogen Peroxide Induced Oxidative Stress
Source: PLoS One. 2013 Jan 28;8(1):e55110. doi: 10.1371/journal.pone.0055110 (PMC3557267; doi:10.1371/journal.pone.0055110)

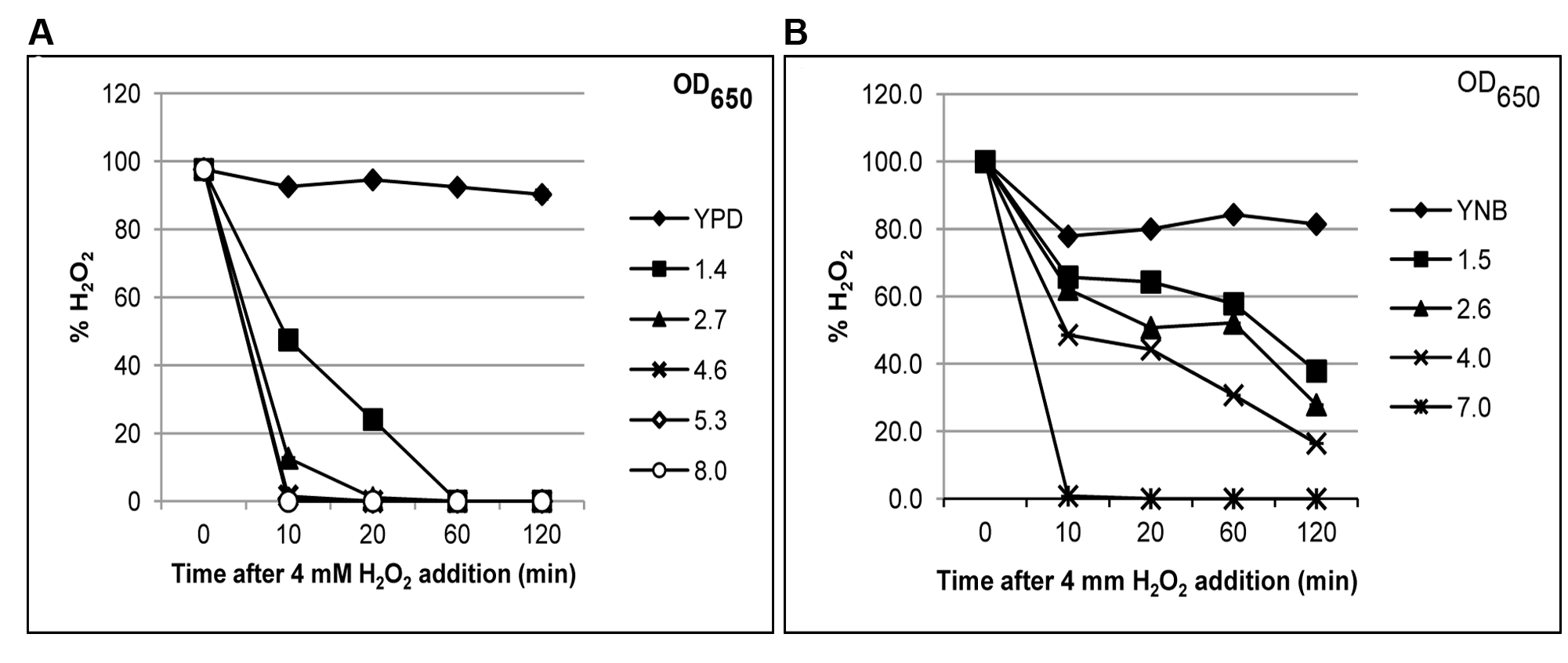

Supplement: Figure S1 — The affect culture density on H2O2 breakdown by C. neoformans cells. A 4 mM of H2O2 was added to cultures at various densities (OD650) growing in YPD (A) and YNB (B). At various time points samples were withdrawn, cells separated by centrifugation and the supernatant was used for H2O2 estimation. The percentage of residual H2O2 was plotted against H2O2 treatment time. Standard bars reflect standard error calculated from three independent experiments. (TIF) [file pone.0055110.s001.tif]

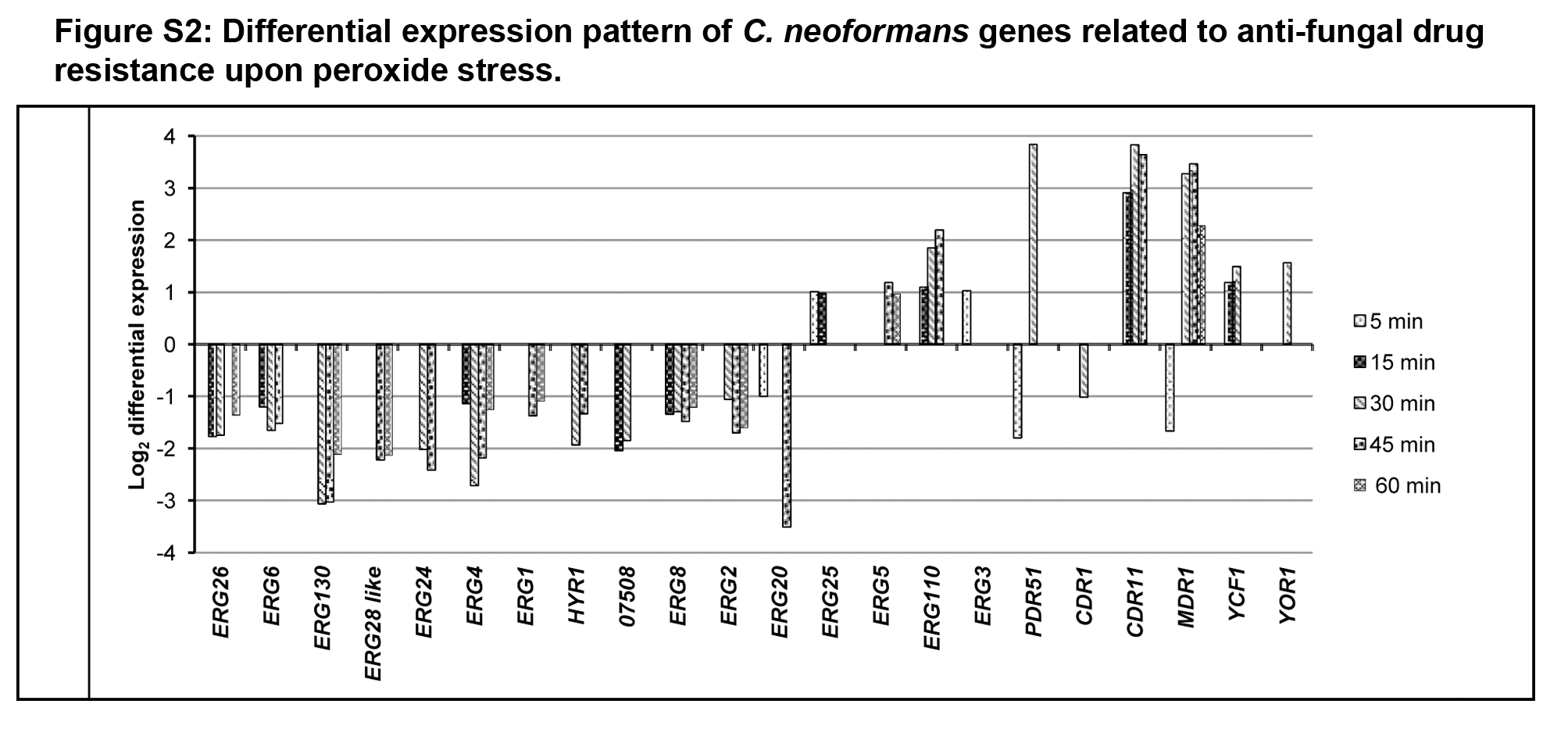

Supplement: Figure S2 — Differential expression pattern of C. neoformans genes related to anti-fungal drug resistance upon peroxide stress. (TIF) [file pone.0055110.s002.tif]
