# Supplementary material for: Global Transcriptome Profile of Cryptococcus neoformans during Exposure to Hydrogen Peroxide Induced Oxidative Stress
Source: PLoS One. 2013 Jan 28;8(1):e55110. doi: 10.1371/journal.pone.0055110 (PMC3557267; doi:10.1371/journal.pone.0055110)
Supplement: Table S2 — Gene ontology enrichment analysis of the differentially expressed probes at various time points during H2O2 treatment. (PDF) [file pone.0055110.s004.pdf]

Table S2: Gene ontology enrichment analysis of the differentially expressed probes at various time points during H_2O_2 treatment.

Time post treatment	Biological Process	P value
5 min	Transport, metabolic process, oxidation reduction, transmembrane transport, cellular response to stress, response to drug, transcription, DNA-dependent, ribosome biogenesis and assembly, regulation of transcription, rRNA processing, pathogenesis, amino acid biosynthetic process, phosphorylation, protein transport, translation, filamentous growth.	2.20^{-16}
15 min	Transport, metabolic process, oxidation reduction, transmembrane transport, cellular response to stress, response to drug, transcription: DNA-dependent, ribosome biogenesis and assembly, regulation of transcription, rRNA processing, pathogenesis, amino acid biosynthetic process, phosphorylation, protein transport, translation, filamentous growth, electron transport chain, signal transduction, cell cycle.	2.20^{-16}
30 min	Transport, metabolic process, oxidation reduction, response to drug, transcription-DNA-dependent, regulation of transcription, transmembrane transport, protein transport, ribosome biogenesis and assembly, rRNA processing, cellular response to stress, cell cycle, regulation of mitotic cell cycle, intracellular protein transport, filamentous growth, signal transduction, vesicle-mediated transport, pathogenesis, protein-phosphorylation, mRNA processing, cell division, response to DNA damage stimulus, proteasomal ubiquitin-dependent protein catabolic process, hyphal growth, translation, DNA repair, RNA splicing, ER to Golgi vesicle-mediated transport, ion transport, protein folding, meiosis, chromatin modification, fungal-type cell wall organization and biogenesis, lipid metabolic process, nuclear mRNA splicing, tRNA processing, endocytosis, proton transport, protein amino acid dephosphorylation, sporulation, electron transport chain, small GTPase mediated signal transduction, vacuolar acidification, DNA replication, GTP catabolic process, carbohydrate metabolic process, amino acid biosynthetic process, regulation of translation, replicative cell aging, ribosomal large subunit assembly and maintenance.	2.20^{-16}
45 min	Transport, metabolic process, oxidation reduction, translation, transmembrane transport, response to drug, cell cycle, cellular response to stress, protein transport, transcription-DNA-dependent, regulation of transcription, cell division, response to DNA damage stimulus, DNA repair, amino acid biosynthetic process, mitosis, pathogenesis, phosphorylation, vesicle-mediated transport, intracellular protein transport, filamentous growth, ion transport, ATP catabolic process, hyphal growth, rRNA processing, meiosis, protein folding, ribosome biogenesis and assembly, translational initiation, DNA replication, carbohydrate metabolic process, mitochondrial translation, signal transduction, ER to Golgi vesicle-mediated transport, regulation of mitotic cell cycle, protein amino acid phosphorylation, mRNA processing, GTP catabolic process, RNA splicing, lipid metabolic process, fungal-type cell wall organization and biogenesis, chromatin modification, proton transport, small GTPase mediated signal transduction, translational elongation, nuclear mRNA splicing, sporulation, endocytosis, ergosterol biosynthetic process, glutamine metabolic process.	2.20^{-16}
60 min	Metabolic process, transport, oxidation reduction, translation, cell cycle, transmembrane transport, regulation of transcription-DNA-dependent, cell division, response to DNA damage stimulus, transcription, DNA-dependent DNA repair, cellular response to stress, response to drug, mitosis, protein transport, amino acid biosynthetic process, meiosis, DNA replication, carbohydrate metabolic process, filamentous growth, pathogenesis, rRNA processing, hyphal growth, phosphorylation, regulation of mitotic cell cycle, signal transduction, intracellular protein transport, ribosome biogenesis and assembly, ATP catabolic process, electron transport chain, lipid biosynthetic process, vesicle-mediated transport, ion transport, DNA replication initiation and GTP catabolic process.	2.20^{-16}
