# Supplementary material for: Global Transcriptome Profile of Cryptococcus neoformans during Exposure to Hydrogen Peroxide Induced Oxidative Stress
Source: PLoS One. 2013 Jan 28;8(1):e55110. doi: 10.1371/journal.pone.0055110 (PMC3557267; doi:10.1371/journal.pone.0055110)
Supplement: Table S5 — List of C. neoformans differentially expressed genes assigned to oxidation-reduction functional category that exhibited persistent induction or repression for a minimum of three time points during H2O2 induced oxidative stress. (PDF) [file pone.0055110.s007.pdf]

Table S5: List of *C. neoformans* differentially expressed genes assigned to oxidation-reduction functional category that exhibited persistent induction or repression for a minimum of three time points during H_2O_2 induced oxidative stress.

			Time after H_2O_2 addition (min)					
**GENE ID**	**ANNOTATION**	**NAME**	**5**	**10**	**15**	**30**	**45**	
CNAG-01846	Pyridine nucleotide-disulphide oxidoreductase							
CNAG-00462	Flavoprotein-ubiquinone oxidoreductase	*CIR2*						
CNAG-02266	NADH ubiquinone oxidoreductase							
CNAG-04325	Aromatic ring-opening dioxygenase							
CNAG-02580	No Information							
CNAG-02722	Aldo/keto reductase family							
CNAG-04351	Aldehyde dehydrogenase family	*ALD52*						
CNAG-00984	short chain dehydrogenase	*FOX22*						
CNAG-03674	Dehydrogenase E1 component	*KGD1*						
CNAG-03589	2Fe-2S iron-sulfur cluster binding domain	*YAH1*						
CNAG-06169	Alcohol dehydrogenase GroES-like domain	*BDH1*						
CNAG-02933	Zinc-binding dehydrogenase	*ZTA1*						
CNAG-05842	Cytochrome P450	*ERG110*						
CNAG-00575	Catalase (CAT3) no in Bahns data	*CTA12*						
CNAG-02868	Indoleamine 2,3-dioxygenase	*BNA2*						
CNAG-01542	No Information	*0*						
CNAG-06638	Malic enzyme, NAD binding domain	*MAE1*						
CNAG-06383	Cytochrome b5-like Heme/Steroid binding	*CYB25*						
CNAG-04553	short chain dehydrogenase							
CNAG-03517	Pyridine nucleotide-disulphide oxidoreductase	*NDE2*						
CNAG-03874	Pyridine nucleotide-disulphide oxidoreductase	*AIF12*						
CNAG-02577	Fatty acid hydroxylase	*SCS7*						
CNAG-02815	FAD dependent oxidoreductase	*GUT2*						
CNAG-03834	Sterol desaturase	*SUR2*						
CNAG-04687	Fatty acid desaturase	*OLE1*						
CNAG-00654	No Information	*SRX1*						
CNAG-05090	Zinc-binding dehydrogenase	*ZTA13*						
CNAG-03238	No Information	*0*						
CNAG-03389	Taurine catabolism dioxygenase TauD	*JLP1*						
CNAG-00993	Ubiquinone biosynthesis protein COQ7	*CAT5*						
CNAG-05440	Pyridoxamine 5'-phosphate oxidase	*PDX3*						
CNAG-03266	lactate/malate dehydrogenase	*MDH1*						
CNAG-01914	No Information mitochondrial protein	*YLR290*						
CNAG-02830	Ergosterol biosynthesis	*ERG4*						
CNAG-06917	AhpC/TSA family	*TSA3*						
CNAG-00879	Glut/Leu/Phe/Val dehydrogenase	*GDH2*						
CNAG-04488	short chain dehydrogenase	*IFA38*						
CNAG-07749	short chain dehydrogenase 2.3xe-16	*TSC10*						
CNAG-01120	2-oxoacid dehydrogenases acyltransferase	*LAT1*						
CNAG-04969	UDP-glucose/GDP-mannose dehydrogenase	*UDG1*						
CNAG-06012	Oxidoreductase FAD-binding domain	*CBR1*						
CNAG-06764	short chain dehydrogenase	*FOX21*						
CNAG-05059	Transketolase, pyridine binding domain	*PDB1*						
CNAG-00012	Oxidoreductase family, NAD-binding							
CNAG-07660	Dehydrogenase E1 component	*PDA1*						
CNAG-03322	NAD dependent epimerase/dehydratase family	*GAL1*						

CNAG-04605	3-beta hydroxysteroid dehydrogenase/family	*ERG26*						
CNAG-02590	NAD bind 6-phosphogluconate dehydrogenase	*0*						
CNAG-04652	Zinc-binding dehydrogenase							
CNAG-02099	Acyl transferase domain	*FAS1*						
CNAG-02208	Ribonucleotide reductase, barrel domain	*RNR1*						

down-regulation

up-regulation

Potential homologs in *S. cerevisiae* predicted to be located to mitochondria.

Each gene has been named after identification of its potential ortholog in *S. cerevisiae* genome. Mitochondrial location of *S. cerevisiae* gene has been obtained from individual gene annotation page at *S. cerevisiae* genome database.
